# High‐Uniformity Threshold Switching HfO_2_‐Based Selectors with Patterned Ag Nanodots

**DOI:** 10.1002/advs.202002251

**Published:** 2020-10-08

**Authors:** Yujia Li, Jianshi Tang, Bin Gao, Wen Sun, Qilin Hua, Wenbin Zhang, Xinyi Li, Wanrong Zhang, He Qian, Huaqiang Wu

**Affiliations:** ^1^ Institute of Microelectronics Beijing Innovation Center for Future Chips (ICFC) Tsinghua University Beijing 100084 China; ^2^ Faculty of Information Technology Beijing University of Technology Beijing 100124 China; ^3^ Beijing National Research Center for Information Science and Technology (BNRist) Tsinghua University Beijing 100084 China

**Keywords:** Ag nanodots, high‐uniformity, one‐selector‐one‐resistor (1S1R), selectors, threshold switching

## Abstract

High‐performance selector devices are essential for emerging nonvolatile memories to implement high‐density memory storage and large‐scale neuromorphic computing. Device uniformity is one of the key challenges which limit the practical applications of threshold switching selectors. Here, high‐uniformity threshold switching HfO_2_‐based selectors are fabricated by using e‐beam lithography to pattern controllable Ag nanodots (NDs) with high order and uniform size in the cross‐point region. The selectors exhibit excellent bidirectional threshold switching performance, including low leakage current (<1 pA), high on/off ratio (>10^8^), high endurance (>10^8^ cycles), and fast switching speed (≈75 ns). The patterned Ag NDs in the selector help control the number of Ag atoms diffusing into HfO_2_ and confine the positions to form reproducible filaments. According to the statistical analysis, the Ag NDs selectors show much smaller cycle‐to‐cycle and device‐to‐device variations (*C*_V_ < 10%) compared to control samples with nonpatterned Ag thin film. Furthermore, when integrating the Ag NDs selector with resistive switching memory in one‐selector‐one‐resistor (1S1R) structure, the reduced selector variation helps significantly reduce the bit error rate in 1S1R crossbar array. The high‐uniformity Ag NDs selectors offer great potential in the fabrication of large‐scale 1S1R crossbar arrays for future memory and neuromorphic computing applications.

## Graphical abstract

A high‐performance selector is fabricated by patterning highly ordered Ag nanodots to control the formation of reproducible filaments. The selector exhibits low leakage current <1 pA, high on/off ratio >10^8^, high endurance >10^8^ cycles, fast switching speed ≈75 ns, and excellent uniformity with *C*_V_ < 10%. It is further integrated into one‐selector‐one‐resistor structure to demonstrate the feasibility of large‐scale array operation.

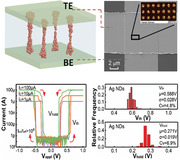

## 1 Introduction

Due to its high speed, small cell size, and low cost, resistive random‐access memory (RRAM) has been extensively studied as a promising candidate for high‐density memory technology, large‐scale neuromorphic computing system, wearable electronics, and Internet of Things (IoTs).^[^ ^1^, ^2^, ^3^, ^4^, ^5^ ^]^ A crossbar array, which takes the advantage of the simple structure and small footprint of RRAM, is an optimal structure for high‐density integration.^[^ ^6^, ^7^, ^8^, ^9^ ^]^ However, the undesired parasitic leakage paths through unselected cells limit the array size and increase the power consumption in operating such crossbar array.^[^ ^10^, ^11^, ^12^ ^]^ To suppress this sneak path current, one‐selector‐one‐resistor (1S1R) structure is widely designed by connecting a selector device with a RRAM device in series. In the literature, many different selector devices such as mixed‐ionic‐electronic‐conduction (MIEC),^[^ ^13^, ^14^, ^15^, ^16^, ^17^ ^]^ tunneling diodes,^[^ ^18^, ^19^ ^]^ ovonic threshold switching (OTS) devices,^[^ ^20^, ^21^, ^22^, ^23^ ^]^ and metal–insulator transition (MIT) or Mott insulators^[^ ^24^, ^25^, ^26^, ^27^, ^28^ ^]^ have been studied. Among them, threshold switching (TS) selectors based on electrochemical metallization (ECM) filaments have attracted considerable attention due to their potential advantages, such as simple structure, large on/off ratio, and low leakage current.^[^ ^29^, ^30^, ^31^, ^32^, ^33^, ^34^, ^35^, ^36^ ^]^ In the fabrication of TS selectors, Ag or Cu is commonly used as the active metal ions to form conductive filaments in dielectric.^[^ ^29^, ^35^, ^36^, ^37^ ^]^ HfO_2_, as a typical transition‐metal oxide, is usually used as the active switching dielectric because of its high dielectric constant, excellent chemical and thermal stability, and complementary metal–oxide–semiconductor (CMOS) compatibility.

Uniformity, as a key metric of selectors, has critical impacts on the performance of large‐scale 1S1R arrays, as shown in Figure S1 (Supporting Information). Nevertheless, the device uniformity of existing TS selectors is still far from satisfactory, owing to the large variability in the size and distribution of the stochastic conductive filaments, which hinders large‐scale integration in practical crossbar arrays. There have been several attempts to investigate and reduce the variation of TS selectors. For example, Park et al. proposed an HfO*_x_*:N‐based ECM selector,^[^ ^38^, ^39^ ^]^ where the nitrogen ions in the HfO*_x_*:N layer assist confining the path of metallic filaments, which helps improve the device uniformity. However, the HfO*_x_*:N selectors show unidirectional switching characteristics, and also slow speed with typical switching time longer than 1.5 µs. Some previous works have reported that the device characteristics of RRAM can be improved by using NDs,^[^ ^40^, ^41^, ^42^, ^43^ ^]^ such as ordered metal/oxide/metal NDs and Ag_2_S/Ag NDs for nanoscale RRAM. In our previous study, an ultrathin anodic aluminum oxide (AAO) template was used as a pattern mask to fabricate Ag NDs as the active metal source in the selector.^[^ ^44^ ^]^ This device showed large on/off ratio and low leakage current. However, the threshold voltage of the device is small (≈0.25 V), and the switching speed is not fast enough (≈200 ns). Moreover, this selector showed unsatisfactory uniformity owing to a large variation in the morphology and distribution of Ag NDs, as shown in Figure S2a (Supporting Information). In addition, the AAO template is not suitable for large‐area fabrication and the reproducibility of the AAO template‐patterned Ag NDs is relatively poor. Therefore, it is still a great challenge to make high‐uniformity TS selectors with a convenient and scalable method.

In this study, in order to improve the uniformity of HfO_2_‐based TS selectors, we use e‐beam lithography (EBL) to pattern controllable Ag NDs with high order and uniform size in the cross‐point region. Rapid thermal annealing (RTA) process is further used to facilitate the diffusion of Ag atoms into HfO_2_ for the formation of conductive filaments. The experimental results demonstrate that this fabrication process is controllable and reproducible, and the fabricated Ag NDs‐based selectors exhibit excellent performance, including large on/off ratio over 10^8^, switching speed as fast as 75 ns, and endurance beyond 10^8^ cycles. Furthermore, an in‐depth study on the device uniformity is performed based on extensive measurements and statistical analysis on the data, showing that our Ag NDs‐based selectors have much improved uniformity and less variations than the previous AAO‐patterned and also nonpatterned devices. The effect of the Ag NDs size on the device uniformity is also analyzed. The selector is further integrated with RRAM to constitute a functional 1S1R unit cell, which shows good device performance for SET/RESET operations.

**Figure 1:**
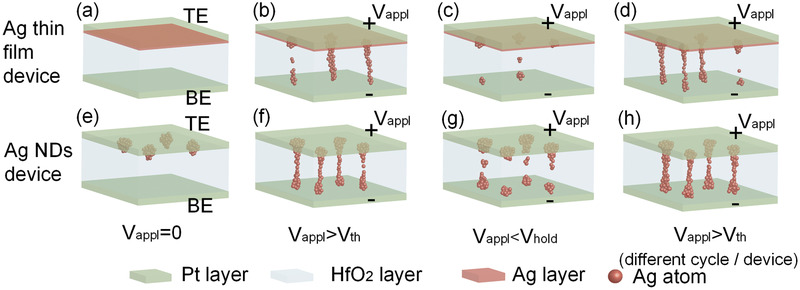
Schematic illustration of the threshold switching process in Ag thin film based selector device: a) initial state, b) filament formation, c) filament rupture, d) repeated operations; and EBL‐patterned Ag NDs based selector device: e) initial state, f) filament formation, g) filament rupture, and h) repeated operations.

The fabrication process of HfO_2_‐based TS selectors with Ag NDs is schematically illustrated in Figure S3 (Supporting Information). A comparison on the switching mechanism of the Ag thin film and NDs based selectors is schematically illustrated in **Figure** **1**. In the Ag thin film device, the position and size of Ag filaments are random, which could lead to a large variation in the selector performance, as shown in Figure 1a–d. However, in the EBL‐patterned Ag NDs device, the Ag NDs with high order and uniform size could accurately determine the positions and morphology of filaments. The subsequent RTA process could drive Ag atoms into the HfO_2_ layer to facilitate the formation of conductive filaments later, as shown in Figure 1e. When a positive voltage is applied on the top electrode, the electric field is locally enhanced in the areas with Ag NDs, so the Ag atoms in these regions are easier to be ionized into Ag ions (Ag → Ag^+^ + e^−^), diffusing toward the bottom electrode and forming metallic filaments between the top and bottom electrodes.^[^ ^45^, ^46^, ^47^ ^]^ As a result, the selector device turns to a low‐resistance state (LRS), as shown in Figure 1f. Due to the small amount of Ag ions in the device, the Ag filaments are thin and unstable. Spontaneous rupture of the filaments occurs immediately after the removal of the applied voltage, which turns the selector to a high‐resistance state (HRS), where Ag atoms form clusters on the trace of filaments, as shown in Figure 1g. In different operation cycles or different devices, Ag filaments tend to form at the same positions and the formed filaments would have similar morphology thanks to the highly ordered Ag NDs, as shown in Figure 1h. As a comparison, the switching mechanism of the AAO template device is also shown in Figure S2b–e (Supporting Information). Overall, the Ag NDs based selectors are expected to show much smaller variation and higher uniformity compared with Ag thin film and AAO template devices.

**Figure 2:**
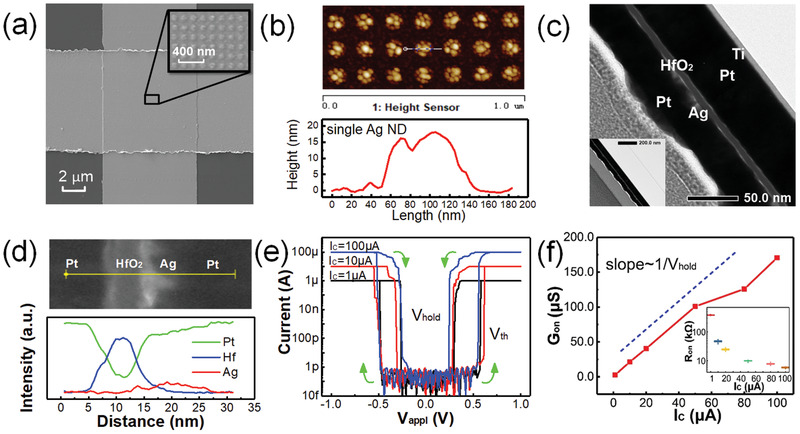
a) SEM image showing the top view of the Ag NDs based selector. Scale bar, 2 µm. b) AFM image showing the surface topology. c) TEM image of the device cross section. d) EDS line‐scan profiles of Pt, Hf, and Ag elements along the yellow line in the TEM image. e) Typical current–voltage (*I*–*V*) curves for the Ag NDs based selector under different compliance current conditions. f) Device on‐state conductance (*G*_on_) as a function of the compliance current (*I*_C_). The inset plots the device on‐state resistance (*R*_on_) as a function of *I*_C_.

**Figure** **2**a shows a top‐view scanning electron microscope (SEM) image of the Ag NDs based selector. The ordered NDs can be seen in the enlarged image. Figure 2b is an atomic force microscope (AFM) image of the Ag NDs. Ag NDs are arranged neatly and have similar morphology. The diameter of the Ag NDs is about 50 nm, and the thickness is about 15 nm. It is observed that the thin Ag layer is not continuous in the NDs. Transmission electron microscope (TEM) image of the device cross section is shown in Figure 2c. Such device structure allows us to accurately control the amount of Ag atoms diffusing into HfO_2_ and predefine the positions to form relatively reproducible filaments. Figure 2d shows the energy‐dispersive X‐ray spectroscopy (EDS) line‐scan profiles of the selector film stack, indicating that certain amount of Ag ions diffuse into the HfO_2_ stack after the RTA process at 400 °C for 30 s. Figure 2e shows a typical current–voltage (*I*–*V*) curve of the Ag NDs based selector under voltage sweeps between −1 and 1 V. The measurements are carried out under different compliance current (*I*_C_) values from 1 to 100 µA. When the applied voltage (*V*_appl_) is increased above the threshold voltage (*V*_th_), the device turns to LRS and the current rapidly reaches *I*_C_. The device turns to HRS spontaneously from its on‐state as *V*_appl_ drops below the hold voltage (*V*_hold_). The *V*_th_ and *V*_hold_ under different *I*_C_ are similar. This Ag NDs based selector shows bidirectional switching characteristic, and the device on/off ratio is more than 10^8^ at the maximum compliance current of 100 µA. Figure 2f shows that the on‐state conductance (*G*_on_), defined as the maximum conductance (*I*_C_/*V*_hold_) at LRS, scales linearly with *I*_C_, which again suggests that the *V*_hold_ values are roughly the same under different *I*_C_. In addition, the Ag NDs based selector was remeasured after four months as shown in Figure S4 (Supporting Information), showing excellent device stability over time.

**Figure 3:**
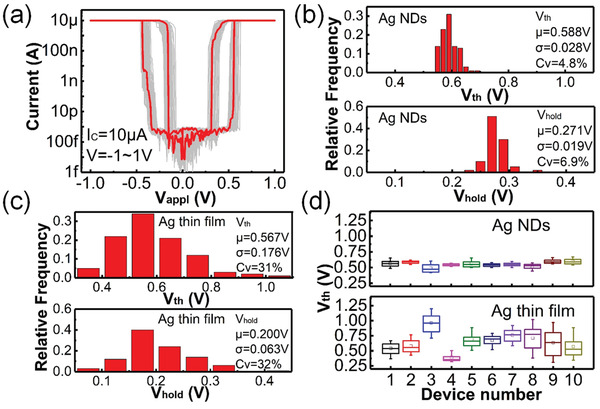
a) Bidirectional *I*–*V* curves for a typical Ag NDs based selector in 100 cycles of DC voltage sweep. b) Statistical distributions of switching voltages (+*V*_th_, +*V*_hold_) for the Ag NDs based selector (the sample size *n* = 100, and the values of *μ* and *σ* are given in the plot). c) Statistical distributions of switching voltages (+*V*_th_, +*V*_hold_) for the Ag thin film selector (the sample size *n* = 100, and the values of *μ* and *σ* are given in the plot). d) Device‐to‐device distributions of *V*_th_ for the Ag NDs selectors and Ag thin film devices.

Repeatable bidirectional volatile switching is observed for the Ag NDs based selector, as shown in **Figure** **3**a, where the device shows no apparent degradation after 100 cycles. In order to evaluate the variability of the Ag NDs based selector, the distributions of *V*_th_ and *V*_hold_ (positive loops) from consecutive 100 cycles are analyzed, as shown in Figure 3b. It can be observed that both *V*_th_ and *V*_hold_ roughly follow a normal distribution. In addition to the standard deviation *σ* and mean value *μ*, another coefficient is defined as *C*_V_ = *σ*/*μ* to evaluate the variation. The *C*_V_ for *V*_th_ and *V*_hold_ are 4.8% and 6.9%, respectively. The distributions of *V*_th_ and *V*_hold_ of the negative loops are also plotted in Figure S5a,b (Supporting Information). Furthermore, *V*_th_ and *V*_hold_ have a narrow distribution even under a higher current compliance of 100 µA, as shown in Figure S6 (Supporting Information). The results indicate that the *C*_V_ for *V*_hold_ is larger than that of *V*_th_, which is determined by the different mechanisms of the selector turn‐on and turn‐off processes (see Section S1, Supporting Information). Figure 3c shows the statistical distributions of *V*_th_ and *V*_hold_ for the Ag thin film selector on the same scale. In comparison, the Ag thin film device without Ag NDs shows a much larger variation, and the *C*_V_ for *V*_th_ and *V*_hold_ are up to 31% and 32%, respectively. Figure 3d compares the device‐to‐device variation for the selectors with Ag NDs and Ag thin film. Ten devices are randomly selected from each type of selectors and tested for 100 cycles of DC *I*–*V* sweep. All devices with Ag NDs have shown repeatable threshold switching with a *V*_th_ mean value ≈0.6 V that fluctuates less than 0.1 V. However, the mean *V*_th_ of Ag thin film devices spanning from 0.4 to 0.9 V shows a much larger device‐to‐device variation. *C*_V_ of Ag NDs selectors shows a narrower distribution compared with the Ag thin film devices and also AAO template device, as shown in Figure S7a,b (Supporting Information). In addition, the *C*_V_ for *V*_th_ and *V*_hold_ of selector devices with different sizes of Ag NDs, different number of Ag NDs as well as different device areas are shown in Figure S8 (Supporting Information). Our results clearly demonstrate that the uniformity of Ag NDs based selectors can be greatly enhanced by optimizing the filament morphology.

**Figure 4:**
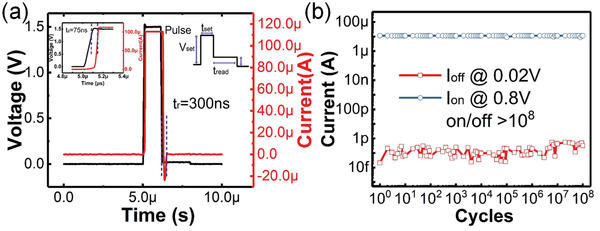
Pulse measurements results of the Ag NDs based selector device. a) The selector switches on within 75 ns (*t*_d_) and returns to HRS within 300 ns (*t*_r_). b) Endurance test of the selector with over 10^8^ cycles.

Furthermore, pulse measurements are performed on the Ag NDs based selectors to study the switching dynamics. The measurement setup used for this experiment is described in the Experimental Section. The voltage pulse waveform is shown in the inset of **Figure** **4**a. *V*_set_ and *t*_set_ are the amplitude and pulse width of SET pulse, respectively. In order to turn the selector to LRS in a short delay time (*t*_d_), *V*_set_ is chosen to be larger than *V*_th_. *V*_read_, which is smaller than *V*_hold_, is the amplitude of the read pulse, and *t*_read_ the pulse width. When *V*_read_ is applied, the device turns to HRS spontaneously after a finite relaxation time (*t*_r_). The dynamic response observed in these devices is shown in Figure 4a. With *V*_set_ = 1.5 V and *t*_set_ = 1 µs, the Ag NDs based selector exhibits a transition from HRS to LRS within 75 ns (*t*_d_). On the other hand, *t*_r_ is about 300 ns when *V*_read_ = 0.02 V is applied. In addition, endurance measurements are performed. The device is repeatedly switched to the on‐state with SET pulse of *V*_set_ = 0.8 V and *t*_set_ = 10 µs, and then turned to off‐state with read pulse of *V*_read_ = 0.02 V and *t*_read_ = 10 µs. The on‐state current (*I*_on_) is limited to 10 µA. As shown in Figure 4b, the Ag NDs based selector exhibits a high endurance of over 10^8^ cycles.

**Figure 5:**
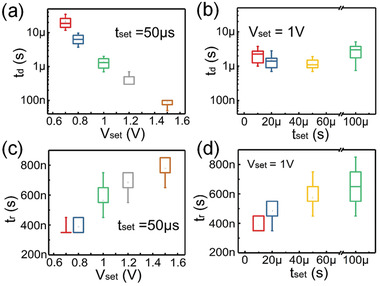
a) The delay time (*t*_d_) distribution dependence on the voltage pulse amplitude (*V*_set_). b) The relationship between the variation of delay time *t*_d_ and the pulse width (*t*_set_). c) The relaxation time (*t*_r_) distribution as a function of the pulse amplitude *V*_set_. d) The relaxation time *t*_r_ dependence on the voltage pulse width *t*_set_.

In order to study the switching speed of our devices, the distributions of *t*_d_ and *t*_r_ are systematically studied under different SET pulse amplitudes and widths. Consecutive pulses are applied for each case to obtain the statistical distributions. The on‐state current is limited to 10 µA. The data are presented in **Figure** **5**. It is found that *t*_d_ decreases exponentially as a function of the pulse amplitude *V*_set_ as shown in Figure 5a, which agrees well with the field‐induced nucleation theory,^[^ ^48^ ^]^ where *t*_d_ can be described as

(1) $$\begin{array}{c} t_{d}=t_{0}\exp\;\left(\frac{w_{0}\alpha^{3/2}E_{0}d}{{kTV}_{\mathrm{set}}}\right) \end{array}$$

Here, *W*_0_ is the nucleation barrier energy at zero field, *α* is a geometric factor, *E*_0_ is the voltage acceleration factor independent of the external field or temperature, *d* is the device dielectric thickness, *T* is ambient temperature, *k* is the Boltzmann constant, and *V*_set_ is applied voltage. Equation (1) also shows that there is no obvious correlation between *t*_d_ and the pulse width *t*_set_, as experimentally confirmed in Figure 5b. On the contrary, *t*_r_ exhibits a clear dependence on *V*_set_ and *t*_set_ as shown in Figure 5c,d. When *V*_set_ and *t*_set_ are large, there is a high electric field in the dielectric and the filament formation duration is long. So the filaments are thick and hard to rupture during the read pulse, making *t*_r_ increase significantly with *V*_set_ and *t*_set_ . Our results also suggest that future optimizations can be done on the pulse condition as well as material stack to further improve the selector switching speed.

**Figure 6:**
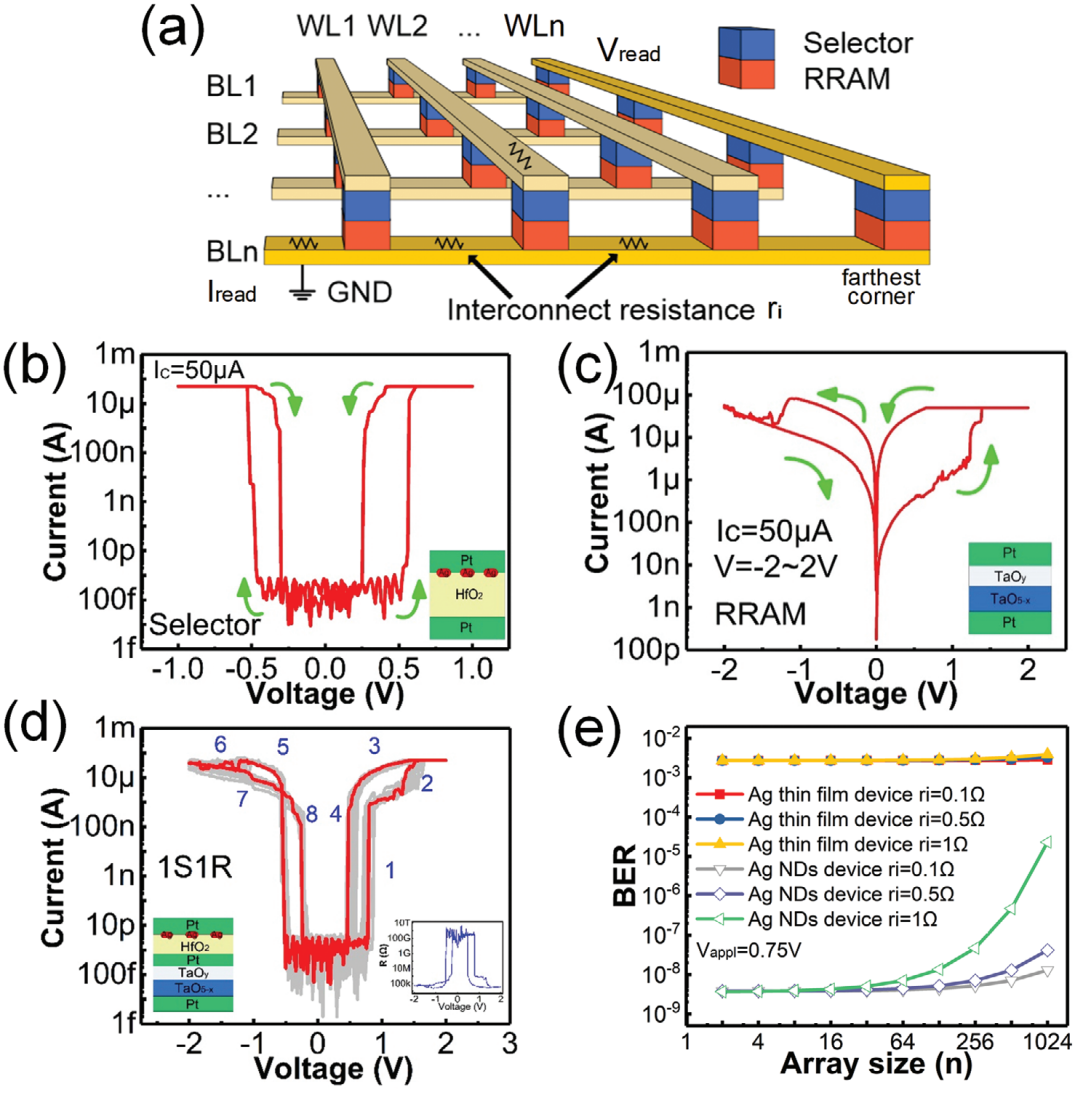
a) Schematic diagram of 1S1R crossbar array structure with interconnect resistance (*r*_i_). b) Typical *I*–*V* curves for the Ag NDs based selector. c) Typical *I*–*V* curves for the Pt/TaO*_y_*/Ta_2_O_5−_ *_x_*/Pt RRAM. d) Typical *I*–*V* curves measured from the integrated 1S1R device. e) The relationship between the estimated BER and the array size (*n*) under an applied read voltage of 0.75 V and different interconnect resistance *r*_i_.

In practical applications, a selector device is usually connected in series with a RRAM to constitute the cell structure in 1S1R crossbar to achieve high‐density integration and large capacity. The schematic diagram of 1S1R crossbar array structure is shown in **Figure** **6**a. In this study, we connect an Ag NDs based selector and a Pt (50 nm)/TaO*_y_* (40 nm)/Ta_2_O_5−_ *_x_* (20 nm)/Pt (50 nm) RRAM in series to form 1S1R structure. Figure 6b,c shows the *I*–*V* switching curves for the selector and the RRAM, respectively. Figure 6d shows the current response from DC voltage sweep for the integrated 1S1R device. The corresponding resistance–voltage (*R*–*V*) curve is shown in the inset of Figure 6d. At the beginning, the RRAM and selector devices are both at HRS (off‐state), and most of the voltage drop is on the Ag NDs based selector because of its large off‐state resistance (>10^9^ Ω). As the applied voltage increases, the selector first turns to LRS (state 1) followed by the SET transition of the RRAM device at ≈1.3 V (state 2). When the voltage is swept back to zero, the RRAM device remains in the LRS (state 3) and the Ag NDs selector turns to HRS (state 4). Similarly, during the negative voltage sweep, the selector turns on at around −0.5 V and then the RRAM is reset to HRS gradually. As we can see, our results successfully demonstrate the proper operation of 1S1R cell by integrating Ag‐HfO_2_ TS selector with Pt/TaO*_y_*/Ta_2_O_5−_ *_x_*/Pt RRAM, and large‐scale 1S1R crossbar arrays can be designed accordingly.

In practical 1S1R array, the variation in the selector switching characteristics, especially *V*_th_ and *V*_hold_, may result in incorrect operations, such as undesired writing during read (i.e., read disturbance). In addition, with the scale down of memory device size in advanced technology nodes, the interconnect resistance (*r*_i_) increases, which would make the above problems even worse. Here, we use our experimentally derived device data for selectors with and without Ag NDs, and carry out array simulations with different array sizes. Figure 6e shows the relationship between the BER during reading and the array size under a read voltage of 0.75 V applied on one end of the word line. The influence of the variability of different selector devices and interconnect resistance is considered. The 1S1R array with Ag NDs selector shows a much lower BER, especially for larger array size (e.g., >64 × 64) and higher interconnect resistance (e.g., *r*_i_ > 1 Ω). The simulation details are shown in Section S2 (Supporting Information). This result also highlights the importance to reduce the device variation using Ag NDs for implementing large‐scale 1S1R arrays.

In summary, we have demonstrated an Ag NDs‐HfO_2_ TS selector with high uniformity by optimizing filaments morphology. EBL is used to pattern controllable Ag NDs in the cross‐point region to regulate the morphology and also limit the amount of Ag atoms for filaments formation. Microscopic inspections, including SEM, AFM, and TEM, show that Ag NDs of uniform size are highly ordered, which can form predefined and reproducible filaments during operation. Electrical measurements manifest that the Ag NDs based selector has a high on/off ratio (more than 10^8^), fast switching speed (75 ns at 1.5 V), and excellent endurance (more than 10^8^ cycles). Statistical analysis confirms that the selectors with Ag NDs show much smaller cycle‐to‐cycle and device‐to‐device variations (*C*_V_ < 10%) compared to those Ag thin film devices (*C*_V_ > 30%) and AAO template devices (*C*_V_ > 15%), and hence exhibit excellent uniformity. A comprehensive comparison of the device characteristics of literature‐reported TS selectors is summarized in Table S1 (Supporting Information). Moreover, the selector is integrated with RRAM to constitute 1S1R structure, which shows good device performance for SET/RESET operations, and the reduced selector variation helps significantly reduce the BER in 1S1R crossbar array. The demonstrated high‐uniformity Ag NDs selectors could pave the road to realize 1S1R crossbar arrays for high‐density memory storage and large‐scale neuromorphic computing.

## 2 Experimental Section

##### Fabrication of EBL‐Patterned Ag NDs Selector

The HfO_2_‐based TS selectors with a device size of 10 × 10 µm^2^ were fabricated on a Si wafer with 200 nm‐thick thermal oxide. First, the bottom electrode was patterned by photolithography followed by sputtering of 5 nm Ti and 50 nm Pt. 8 nm‐thick HfO_2_ dielectric was deposited by atomic layer deposition (ALD) at 250 °C. Subsequently, the Ag NDs with different diameters of 50, 100, and 200 nm were patterned by EBL and deposited by sputtering. 40 nm‐thick Pt top electrode was patterned and deposited using the same technique as the bottom electrode. Then, the sample was etched to open via contacts to the bottom electrodes. Finally, the sample was treated by the RTA process at 400 °C for 30 s. Different Ag and HfO_2_ thicknesses and RTA temperatures were tested to adjust the threshold voltage to an appropriate value that can match with the RRAM device.

##### Electrical Measurements

The DC *I*–*V* sweeps were performed using Keysight B1500A semiconductor device parameter analyzer. Keysight B1530A waveform generator/fast measurement unit (WGFM) and Keysight B1500A semiconductor device parameter analyzer were used to measure *t*_d_ and *t*_r_. Keysight 81160A pulse function arbitrary noise generator, Keysight B1500A semiconductor device parameter analyzer, and Keysight B2201A 14ch low leakage switch mainframe were used to measure the endurance of the Ag NDs based selector. A 20 kΩ resistor was connected in series with the device to limit the ON‐state current.

##### Statistical Analysis

The selector devices were tested for multiple cycles by Keysight B1500A to extract *V*_th_ and *V*_hold_. Histograms were employed to exhibit the distributions of *V*_th_ and *V*_hold_. The sample size (*n*) for each statistical analysis was chosen as 100 unless otherwise indicated. Matlab software was used to calculate the standard deviation *σ* and mean value *μ* for statistical analysis.

## Conflict of Interest

The authors declare no conflict of interest.

## Supporting information

Supporting Information
