# Supplementary material for: High‐Uniformity Threshold Switching HfO2‐Based Selectors with Patterned Ag Nanodots
Source: Adv Sci (Weinh). 2020 Oct 8;7(22):2002251. doi: 10.1002/advs.202002251 (PMC7675059; doi:10.1002/advs.202002251)
Supplement: Supplementary file 1 — Supporting Information [file ADVS-7-2002251-s001.pdf]

WILEY-VCH

© 2020 Wiley-VCH GmbH

ADVANCED SCIENCE
Open Access

Supporting Information

for *Adv. Sci.,* DOI: 10.1002/advs.202002251

High-Uniformity Threshold Switching HfO_2-based Selectors with Patterned Ag Nanodots

Yujia Li, Jianshi Tang, Bin Gao, Sun Wen, Qilin Hua, Wenbin Zhang, Xinyi Li, Wanrong Zhang, He Qian, and Huaqiang Wu**

© 2020 Wiley-VCH GmbH

Supporting Information

High-Uniformity Threshold Switching HfO_2-based Selectors with Patterned Ag Nanodots

Yujia Li, Jianshi Tang, Bin Gao, Sun Wen, Qilin Hua, Wenbin Zhang, Xinyi Li, Wanrong Zhang, He Qian, and Huaqiang Wu**

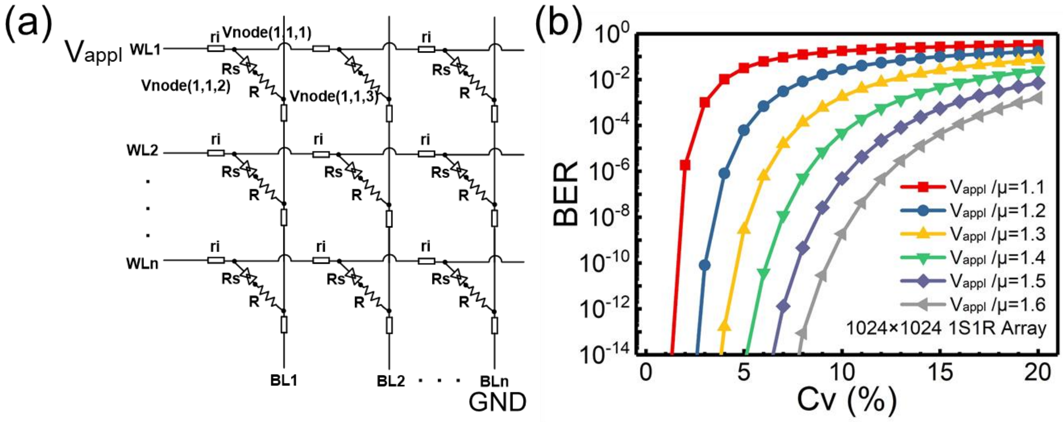

Figure S1. a) Schematic diagram of the simulated 1S1R crossbar array. b) The relationship between the estimated BER of 1S1R array (size of 1024×1024) and C_V (coefficient of variation) of the selector thoreshold voltage V_{th} under different applied voltages V_{appl}.

The impact of selector uniformity on the 1S1R array is investigated in Figure S1. At a certain value of V_{appl}/μ (V_{appl}: applied voltage, μ: mean value of selector V_{th}), the degradation in selector uniformity, namely the increase in C_V of selector V_{th}, leads to a significant rise in BER of the 1S1R array. Reducing the selector variation or increasing the applied voltage can effectively suppress the BER, as shown in Figure S1b. However, a large applied voltage may disturb the RRAM device states and increase the power consumption. Therefore, improving the device unformity of selectors is critical in practical 1S1R crossbar arrays.

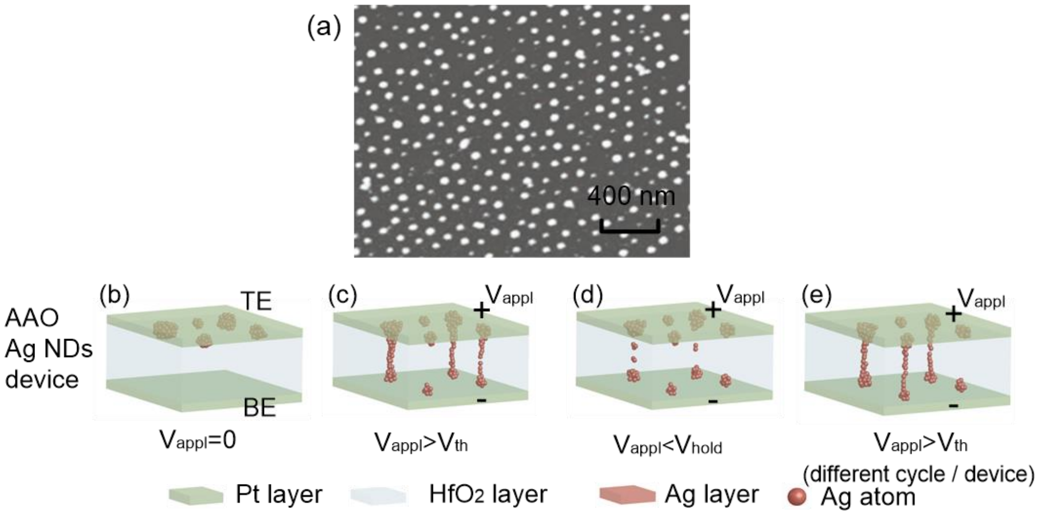

Figure S2. a) SEM image of typical Ag NDs fabricated by AAO template (scale bar: 200 nm). b) ~ e) Schematic illustration of the threshold switching process in AAO template selector device: b) Initial state; c) Filament formation; d) Filament rupture; e) Repeated operations.

The morphology and distribution of Ag NDs fabricated by AAO template shows unsatisfactory uniformity, as shown in **Figure S2**a. As a result, the position and size of Ag filaments are random, especially among different devices, as illustrated in Figure S2b ~ e. This could lead to a large variation in the selector performance.

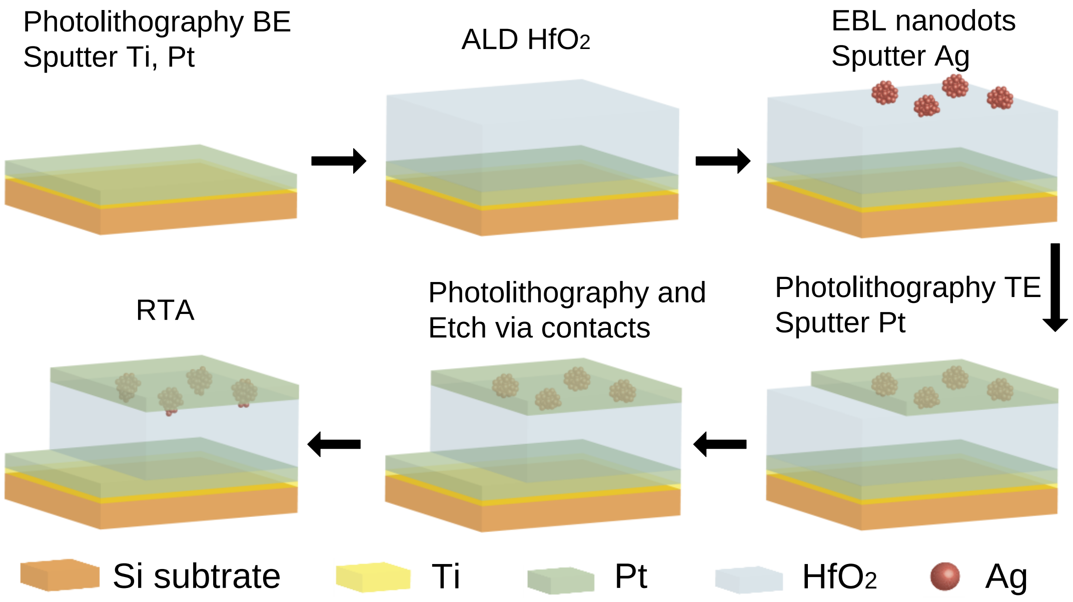

Figure S3. The fabrication process flow of HfO_2-based TS selectors with Ag NDs. The Ag NDs selector were fabricated on a Si wafer with 200 nm-thick thermal oxide. The bottom electrode of 5 nm Ti/50 nm Pt was patterned by photolithography. 8 nm-thick HfO_2 dielectric was deposited by ALD at 250 °C. Then, Ag NDs were patterned by EBL. Subsequently, 40 nm-thick Pt top electrode was patterned and deposited. Via holes were etched to open contacts to the bottom electrode. Finally, the sample was treated by RTA at 400 °C for 30 s.

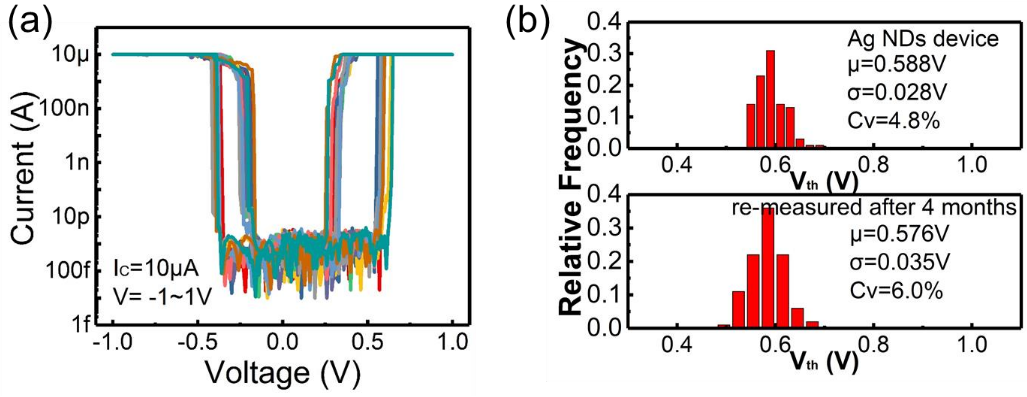

Figure S4. Re-measured results of the Ag NDs selector after 4 months, showing excellent stability over time. a) I–V curves for the Ag NDs selector. b) Statistical distributions of the threshold voltage (V_{th}) for the Ag NDs selectors (the sample size n = 100, and the values of μ and σ are given in the plot).

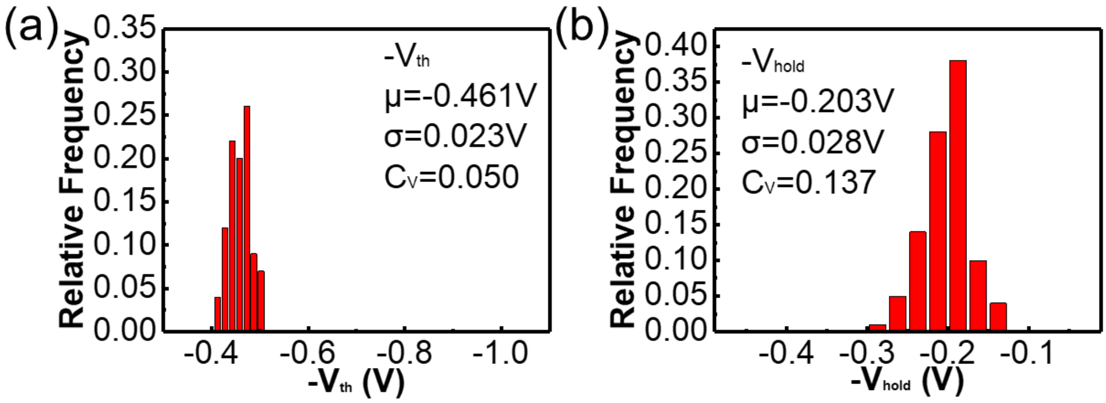

Figure S5. Statistical distributions of a)-V_{th} and b) -V_{hold} (extracted from the negative loops) for the Ag NDs based selectors. The sample size n =100, and the values of μ and σ are given in the plot.

The distributions of V_{th} and V_{hold} of the negative loops are also plotted in **Supporting Information Figure S5a~b**. The C_V for -V_{th} and -V_{hold} are 5% and 13%, respectively.

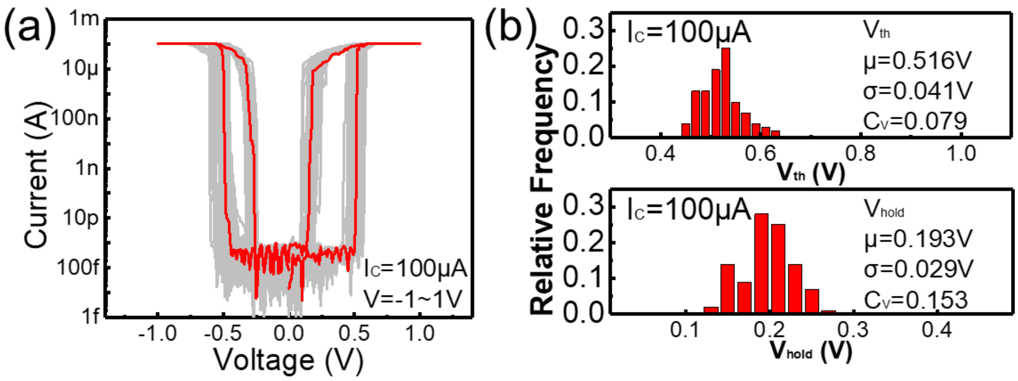

Figure S6. a) Bi-directional I–V curves for a typical Ag NDs-based selector under a compliance current of 100 μA. b) Statistical distributions of switching voltages (Vth, Vhold) for the Ag NDs-based selector. The sample size n =100, and the values of μ and σ are given in the plot.

The cycle to cycle I-V curves of the Ag NDs-based selectors under a compliance current of 100 μA are shown in **Supporting Information Figure S6a**. The statistical distributions of V_{th} and V_{hold} under the compliance current of 100 μA are shown in **Supporting Information Figure S6b.** The C_V for V_{th} and V_{hold} are estimated as 7.9% and 15.4%, respectively. It is shown that the uniformity of Ag NDs selectors device remains high even under a higher current compliance of 100 μA. However, compared with the results under the compliance current of 10 μA, the uniformity of the Ag NDs-based selectors is degraded with increasing compliance current, especially for V_{hold}. This is likely because the diameter and quantity of conductive filaments formed in the dielectric layer increases under a higher compliance current, which intensifies the uncertainty of the morphology of filaments. As a result, the device uniformity deteriorates.

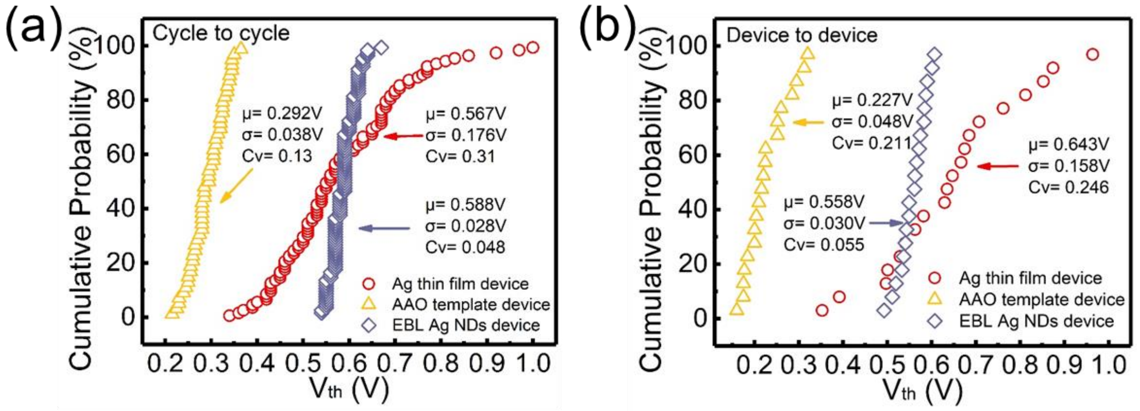

Figure S7. Cumulative probability of V_{th} distributions for the Ag NDs based selectors, Ag thin film devices and AAO template devices: a) cycle-to-cycle variation (the sample size n = 100, and the values of μ and σ are given in the plot) and b) device-to-device variation (the sample size n = 20, and the values of μ and σ are given in the plot).

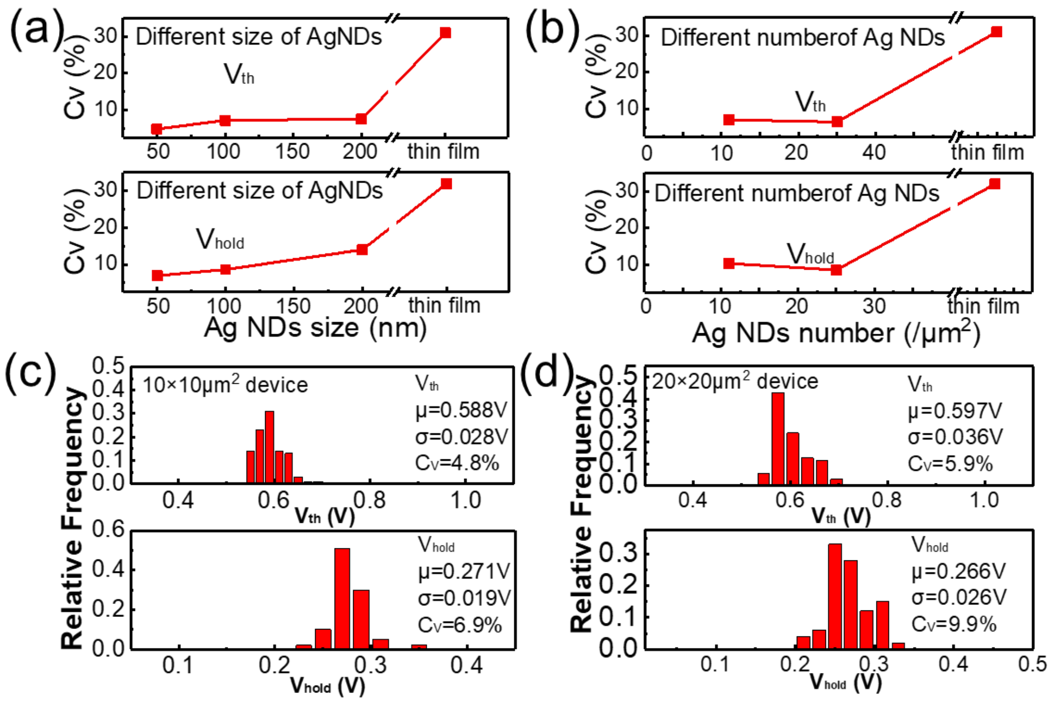

Figure S8. C_V for V_{th} and V_{hold} of selector devices with (a) different sizes of Ag NDs, and (b) different numbers (densities) of Ag NDs (at the same device area of 10×10 μm^2), (c-d) Statistical distributions of V_{th} and V_{hold} for the Ag NDs-based selector with different device areas (at the same density of Ag NDs of 25/μm^2). The sample size n =100, and the values of μ and σ are given in the plot.

Figure S8a illustrates the statistical result of the relationship between the coefficient of variation for V_{th} and V_{hold} and the size of Ag nanodots. the C_V of both V_{th} and V_{hold} increases with the size of Ag NDs. This is consistent with the conclusion made in this work that the confinement of the formation of conductive filaments using Ag NDs helps improve the device uniformity.

In addition, the effects of the number of Ag NDs and the selector device area on V_{th} and V_{hold} are shown in **Figures S8b and S8c~d**, respectively. The test results indicate that the number of Ag NDs and the device area have no obvious effect on the device uniformity, considering that there are actually at least hundreds of Ag NDs for each device. During the switching processes, only a few conductive filaments are expected to form in the dielectric layer of the selector under certain compliance current. When the total number of Ag NDs is

large, the number of Ag NDs and the device area have no obvious effect on the formation of conductive filaments. Future studies may be carried out to limit the number of Ag NDs in each selector in order to systematically study the effect on the device uniformity.

S1. Analysis of the variation in the threshold and hold voltages

In the Ag NDs-based selector device, the formation of conductive filaments is a result of field-induced drift of Ag ions, which can be described by a field-driven, temperature activated migration model. During the rupture process of the conductive filaments, the Ag atoms spontaneously diffuse from the filament region to the electrodes, driven by the tendency of surface energy minimization. The switching process of the device is affected by the two processes above, as described by the following equation[1,2]:

$$\frac{d\phi}{dt} = \frac{d\phi}{dt}\|_{drift} + \frac{d\phi}{dt}\|_{diff} = Ae^{-\frac{E_{bulk} - aqV}{kT}} + \frac{4D_s\gamma\delta^4}{\pi kT}\phi^3 e^{-\frac{E_{surf}}{kT}} \quad (1)$$

where A is a pre-exponential constant, E_{bulk} is the energy barrier for field-driven drift, q is the elementary charge, V is the applied voltage, a is the barrier lowering coefficient, D_s is the surface diffusion coefficient, γ is the surface tension, δ is the inter-atomic distance, k is the Boltzmann's constant, ϕ is the conductive filament diameter, E_{surf} is the energy barrier for surface diffusion and T is the ambient temperature.

During the selector turn-on process, the voltage-controlled filament growth is dominant. Ag filaments tend to form under the same voltage thanks to the highly ordered Ag NDs. So the variation of V_{th} is small. During the selector turn-off process, the spontaneous diffusion of Ag atoms is dominant. The spontaneous diffusion process is not only related to the morphology of conductive filaments, but also affected by environmental conditions surrounding the filaments, as given by **Equation 1**. Therefore, the coefficient of variation for V_{hold} is slightly larger than that of V_{th}.

S2. BER simulation of 1S1R array

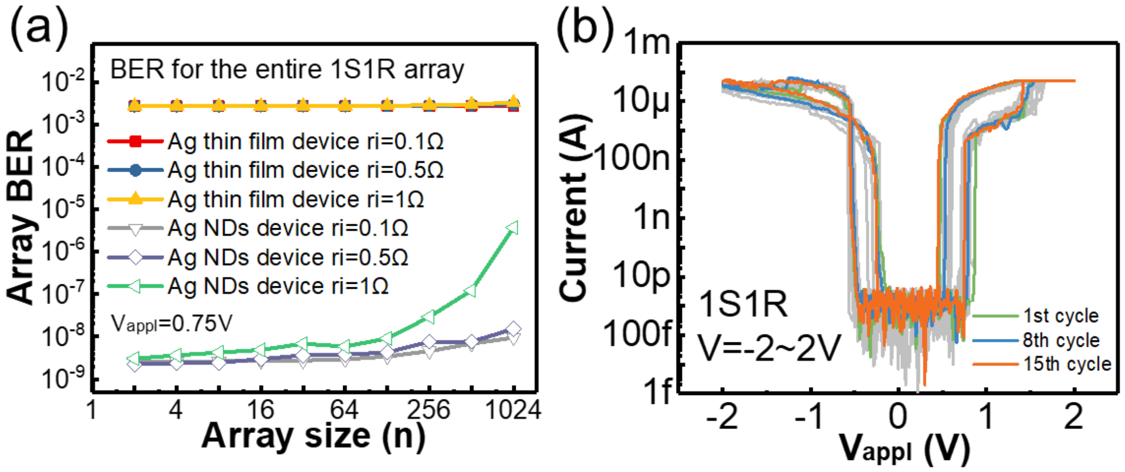

Figure S9. a) BER for the entire 1S1R array in different array sizes. b) I–V curves for 1S1R device of different cycles of DC voltage sweep.

In this work, we model the 1S1R crossbar array using experimentally derived device data for the selectors and RRAMs. The schematic diagram of 1S1R crossbar array structure is shown in **Figure 6a** in the main text. All of the node states in the 1S1R array can be calculated based on the Kirchhoff's Law. In order to study the effect of interconnect resistance (r_i) on the array, all the RRAM devices are set to the low-resistance state (LRS) at the beginning. During the bit error rate (BER) testing, every 1S1R cell in the array is read under a voltage of 0.75 V applied on one end of the word line. If the read current is small (< 0.01 μA), suggesting the selector is not correctly turned on, it is counted as one error bit. To obtain the BER statistically, the read operation is repeated for more than 10^9 times while taking the V_{th} variation of the selectors and the interconnect resistance into consideration. To highlight the influence of device uniformity on the 1S1R array performance, **Figure 6e** in the main text shows the BER for the 1S1R cell located at the farthest corner from the word- and bit-lines (the worst case scenario).[3] In addition, we have also calculated the BER for the entire 1S1R array as shown in **Figure S9a**. Both results confirm that the 1S1R array with Ag NDs-based selectors shows a much lower BER compared to that with Ag thin film ones,

especially for larger array size (e.g., >64×64) and higher interconnect resistance (e.g., $r_i > 1$ Ω). Furthermore, **Figure S9b** (re-drawn from **Figure 6d** in the main text) confirms that the 1S1R cell can still maintain good uniformity after several cycles of SET/RESET operations. As the Ag NDs-based selector has shown a high endurance over 10^8 cycles, it can be expected that the 1S1R array can maintain a low BER after multiple cycles of read and SET/RESET operations.

Table S1. Comparison of literature-reported results on TS selectors.

Material stack	Mode	I_{on}	I_{off}	I_{on}/I_{off}	V_{th}	V_{hold}	C_V of V_{th}	t_d	t_r	Endurance	Ref.
Pt/HfO_2/Cu:HfO_2/Cu	Unipolar	10μA (I_C)	~1pA	10^7	~0.4V	≈0 V	/	/	/	>10^{10}	4
Ag/a-Si:H/Pt	Bipolar	10μA (I_C)	~1pA	10^7	±0.8V	±0.45V	/	/	/	>10^6	5
AgTe/TiN/TiO_2/Pt	Unipolar	100μA (I_C)	~1pA	10^8	0.5V	≈0V	/	/	100ns	/	6
W/Cu_2S/W	Unipolar	10μA (I_C)	~100pA	10^5	0.3V	/	/	/	/	/	7
Ag/HfO_2/SiO_2/p-Si	Unipolar	100μA (I_C)	~10pA	10^7	1.5V	≈0.1V	/	58ns	67ns	10^8	8
Ag/HfO_2/Pt	Unipolar	10μA (I_C)	~1pA	10^7	≈0.3V	≈0.05V	/	30μs	5μs	/	9
Pd/Ag/HfOx/Ag/Pd	Bipolar	100μA (I_C)	~1pA	10^8	±0.4V	±0.1	/	75ns	250ns	10^8	10
Au/Ag NW–PDMS/Au	Bipolar	100μA (I_C)	~1pA	10^{10}	~0.4V	/	/	520ns	110μs	>10^6	11
Ag/TaO_x/TaO_y/TaO_x/Ag	Bipolar	1mA (I_C)	~1pA	10^{10}	tunable	/	/	75ns	500ns	/	12
Ag/HfOx:N/Pt	Unipolar	1mA (I_C)	~10pA	10^8	≈0.2V	≈0.05V	4.2%-17%	1.5μs	5μs	10^6	13
Pt/Ag nanodots/HfO_2/Pt (AAO)	Bipolar	1mA (I_C)	~1pA	10^9	±0.25V	±0.05V	15%	200ns	240ns	>10^8	14
Pt/Ag NDs/HfO_2/Pt (EBL)	Bipolar	100μA (I_C)	~1pA	10^8	±0.6V	±0.25V	4.8%	75ns	300ns	>10^8	This work

$C_V = \sigma/\mu$
I_C, compliance current
t_d, delay time
t_r, relaxation time

Reference:

[1] S. Ambrogio, S. Balatti, D. C. Gilmer, D. Ielmini, *IEEE Trans. Electron Dev.* **2014**, 61, 7, 2378-2386.

[2] W. Wang, M. Laudato, A. Bricalli, E. Covi, Y. Lin, D. Ielmini, *IEEE Trans. Electron Dev.* **2019**, 66, 9, 3802-3808.

[3] Y. Deng, P. Huang, B. Chen, X. Yang, B. Gao, J. Wang, L. Zeng, G. Du, J. Kang, X. Liu, *IEEE Trans. Electron Dev.* **2013**, 60, 2, 719-726.

[4] Q. Luo, X. Xu, H. Liu, H. Lv, T. Gong, S. Long, Q. Liu, H. Sun, W. Banerjee, L. Li, N. Lu, M. Liu, presented at 2015 IEEE Int. Electron Devices Meet. (IEDM), Washington, DC, USA, 7-9 Dec., **2015**.

[5] J. Yoo, J. Woo, J. Song, H. Hwang, *AIP Adv.* **2015**, *5*, 127221.

[6] J. Song, J. Park, K. Moon, J. Woo, S. Lim, J. Yoo, D. Lee, H. Hwang, presented at 2016 IEEE Int. Electron Devices Meet. (IEDM), San Francisco, CA, USA, 3-7 Dec., **2016**.

[7] S. Lim, J. Yoo, J. Song, J. Woo, J. Park, H. Hwang, presented at 2016 IEEE Int. Electron Devices Meet. (IEDM), San Francisco, CA, USA, 3-7 Dec., **2016**.

[8] N. Shukla, B. Grisafe, R. K. Ghosh, N. Jao, A. Aziz, J. Frougier, M. Jerry, S. Sonde, S. Rouvimov, T. Orlova, S. Gupta, S. Datta, presented at *2016 IEEE Int. Electron Devices Meet. (IEDM)*, San Francisco, CA, USA, 3-7 Dec., **2016**.

[9] J. Yoo, J. Park, J. Song, S. Lim, H. Hwang, *Appl. Phys. Lett*. **2017**, 111, 6.

[10] R. Midya, Z. Wang, J. Zhang, S. E. Savel'ev, C. Li, M. Rao, M. H. Jang, S. Joshi, H. Jiang, P. Lin, K. Norris, N. Ge, Q. Wu, M. Barnell, Z. Li, H. L. Xin, R. S. Williams, Q. Xia, J. J. Yang, *Adv. Mater.* **2017**, *29*, 1604457.

[11] M. Wang, W. Wang, W. Leow, C. Wan, G. Chen, Y. Zeng, J. Yu, Y. Liu, P. Cai, H. Wang, D. Ielmini, X. Chen, *Adv. Mater.* **2018**, 30, 1802516.

[12] Y. Sun, X. Zhao, C. Song, K. Xu, Y. Xi, J. Yin, Z. Wang, X. Zhou, X. Chen, G. Shi, H. Lv, Q. Liu, F. Zeng, X. Zhong, H. Wu, M. Liu, and F. Pan, *Adv. Funct. Mater.* **2019**, 29,

1808376.

[13] J. Park, S. Kim, S. Kim, H. Heo, H. Yu, *ACS Appl. Mater. Interfaces.* **2019**, 11, 9182−9189.

[14] Q. Hua, H. Wu, B. Gao, M. Zhao, Y. Li, X. Li, X. Huo, M. Chang, P. Zhou, H. Qian, *Adv. Sci*. **2019**, 6, 1900024.
